# Factors influencing the willingness to participate in medical research: a nationwide survey in Taiwan

**DOI:** 10.7717/peerj.4874

**Published:** 2018-05-31

**Authors:** Hung-En Liu, Ming-Chieh Li

**Affiliations:** 1 Graduate Institute of Law and Interdisciplinary Studies, National Chengchi University, Taipei, Taiwan; 2 Department of Nutrition, Harvard T.H. Chan School of Public Health, Harvard University, Boston, MA, United States of America

**Keywords:** Participant recruitment, Public attitudes, Medical research, Trust in medical research

## Abstract

**Background:**

Participation rate is one of the main challenges medical researchers face. We examined how demographic background and trust in medical research affect the willingness of people to participate in medical research in Taiwan.

**Methods:**

Data from the 2011 Taiwan Genomic Survey (a nationwide representative face-to-face survey) were analyzed. The survey included a vignette of a researcher conducting a clinical trial of an investigative medicinal product, and questions for interviewees regarding their willingness to participate in research after they were informed of the scenario description. A total of 3,159 people, aged 18 to 70 years, were sampled, and 1,538 of them completed the survey. With missing data excluded, a total of 1,389 respondents were included in the final analysis.

**Results:**

About 12 percent of the respondents answered that they would be willing to participate in medical research. Respondents who had college degrees or above and were married or lived with significant others were less likely to participate in medical research. By contrast, male respondents, and respondents whose household family members had biomedicine-related degrees or had one themselves were more likely to participate in medical research. After adjustment for demographic factors, respondents were more likely to participate in medical research if: (1) they expressed trust in doctors conducting medical research; (2) they agreed that doctors would never ask them to join medical research studies that might harm them; (3) they thought that participating in a medical research study would be safe; and (4) they agreed that researchers had no selfish reasons for doing the medical research.

**Discussion:**

Some of our findings, such as the effects of education level and marital status on participation in medical research, are different from most findings of previous studies conducted in other countries. This study is useful for developing strategies to improve participant recruitment. Relevant discussions on research ethics and policies, such as the importance of public trust in medical researchers, could also be based on this study.

## Introduction

Advances in medical care depend on the participation of human participants. However, participation rate is one of the main challenges researchers face (Charlson & Horwitz, 1984). Evidence shows that the long-term participation rate declines gradually (Galea & Tracey, 2007; Morton, Cahill & Hartge, 2005). Low participation creates some concerns and challenges: (1) smaller sample size reduce the accuracy of estimates and statistical power of hypothesis tests, and increase the chance of making Type II errors (Quinn & Keough, 2002); and (2) the results may not be generalizable to the general population (Kho et al., 2009; Kypri et al., 2011; Maclennan et al., 2012; Madigan et al., 2000). To address the aforementioned concerns, understanding potential factors associated with low participation is important.

After reviewing past literatures, we found studies that involved large samples of Asian people to assess their participation in medical research and to determine the influencing factors were limited (Chu et al., 2015; Kim et al., 2008; Sugawara et al., 2015). Therefore, the purpose of this study was to examine how demographic background and trust in medical research affect the willingness to participate in medical research in Taiwan.

## Materials and Methods

### Participants

Ethical approval was not necessary for this study. The data were obtained from the 2011 Taiwan Genomic Survey, conducted by the Survey Research Center of Academia Sinica, Taiwan. The database is publicly accessible (https://srda.sinica.edu.tw/datasearch_detail.php?id=997), but registration is required. It was a nationwide representative survey that investigated Taiwanese understanding and attitudes toward the development of biotechnology. Taiwan has a complete and reliable household registration system (Chen & Yang, 2015; Wu & Young, 1986). In order to obtain a representative sample of the Taiwanese population, the survey used the household registration data in 2010 as its sampling frame. A stratified, three-stage probability, proportional-to-size sampling from across the nation was conducted to select participants aged from 18 to 70 years. A total of 3,159 people were sampled. Face-to-face interviews were conducted with a structured questionnaire by well-trained interviewers (Yu, 2016). Among 3,159 people, 1,538 eligible respondents completed the survey. After data with missing values were excluded, a total of 1,389 respondents were included in the final analysis.

The questionnaire used in the nationwide survey provided some of the information we needed: the willingness to participate in medical research, and demographic descriptions, such as age, sex, education level, employment status, personal income, marital status, religion, and place of residence. We included additional information in our analyses regarding the following items: (1) self-reported health status; (2) whether the participants or their household family members had biomedicine-related degrees; and (3) questions regarding the participants’ trust in medical research.

First, the following scenario describing the background information of a typical medical study in Taiwan was read to the interviewees:

Dr. Chang is conducting pharmaceutical research at an academic institution, and he needs to recruit people to participate in the research (for example, testing for new medication). If Dr. Chang successfully develops the medication, he may then apply for a patent. A pharmaceutical company will produce the medication, and both the pharmaceutical company and their shareholders will profit from the sale of the medication. Dr. Chang invites you to participate in the research. He has explained to you the potential effects and side effects of the medication (such as dizziness, headache, diarrhea, and skin allergies). You will receive NT$500 dollars for participating in the research.

Next, they were questioned regarding their willingness to participate in medical research.

### Statistical analysis

Basic demographic data were summarized as total numbers and percentages for categorical variables. The differences in categorical variables were compared using chi-square tests. Stepwise multiple logistic regression models were used to determine which variables were significantly related to the willingness to participate in medical research. A significance level of 0.05 was required for a variable to remain in the model. All analyses were performed by SAS 9.4 (SAS Institute, Cary, NC, USA).

## Results

About 12 percent of the respondents answered that they would be willing to participate in medical research. Table 1 shows the descriptive statistics of the survey participants. The respondents were divided into two groups: people who were willing to participate in medical research and people who were not. A univariate analysis showed that male participants were more willing to participate in medical research than were female participants (61.82% versus 49.51%).

**Table 1:** Summary of the characteristics of the study participants.

	Willingness to participate in medical research (Total = 1,389)				
	Yes = 165 (11.9%)		No = 1,224 (88.1%)		
Variables	Number	%	Number	%	*P*-value
**Age (years)**					
30 or below	51	30.91	365	29.82	0.79
31–40	30	18.18	259	21.16	
41–50	39	23.64	261	21.32	
51 or above	45	27.27	339	27.70	
**Sex**					
Women	63	38.18	618	50.49	<.01
Men	102	61.82	606	49.51	
**Education level**					
Junior high school or below	49	29.70	294	24.02	0.09
Senior high school or junior college	73	44.24	516	42.16	
College or above	43	26.06	414	33.82	
**Employment status**					
Non-employee	59	35.76	382	31.21	0.35
Part-time job	10	6.06	104	8.50	
Full-time job	96	58.18	738	60.29	
**Personal income**					
NT$20,000 or below	72	43.64	509	41.58	0.87
NT$20,001-NT$50,000	67	40.61	510	41.67	
≥NT$50,000	26	15.76	205	16.75	
**Marital status**					
Other	74	44.85	501	40.93	0.34
Married or living with a significant other	91	55.15	723	59.07	
**Religion**					
No religion	42	25.45	251	20.51	0.53
Folklore religion	48	29.09	414	33.82	
Buddhist/Taoist	62	37.58	464	37.91	
Catholic/Christian	9	5.45	74	6.05	
Other	4	2.42	21	1.72	
**Place of residence**					
Eastern Taiwan and outlying islands	8	4.85	42	3.43	0.46
Southern Taiwan	55	33.33	353	28.84	
Central Taiwan	38	23.03	302	24.67	
Northern Taiwan	64	38.79	527	43.06	
**Self-reported health status**					
Good	135	81.82	1,055	86.19	0.13
Bad	30	18.18	169	13.81	
**Whether the interviewees or their household family members had a biomedicine-related degree**					
No	152	92.12	1,158	94.61	0.20
Yes	13	7.88	66	5.39	

Table 2 presents the results of a stepwise logistic regression analysis of factors that significantly affect the willingness to participate in medical research. The results in the adjusted model revealed that respondents who had college degrees or above (odds ratio (OR) = 0.43, 95% confidence interval (CI) [0.26–0.70]) and were married or lived with significant others (OR = 0.69, 95% CI [0.49–0.99]) were less likely to participate in medical research. On the other hand, respondents who were men (OR = 1.76, 95% CI [1.25–2.47]), and respondents whose household family members had biomedicine-related degrees or had one themselves (OR = 2.24, 95% CI [1.15–4.37]), were more likely to participate in medical research.

**Table 2:** Using stepwise multiple logistic regression to determine the factors influencing the willingness to participate in medical research.

	Odds ratio	95% confidence interval
**Education level**		
Junior high school or below	–	–
Senior high school or junior college	0.75	0.50–1.12
College or above	0.43^*^	0.26–0.70
**Sex**		
Women	–	–
Men	1.76^*^	1.25–2.47
**Marital status**		
Others	–	–
Married or living with a significant other	0.69^*^	0.49–0.99
**Whether the interviewees or their household family members had a biomedicine-related degree**		
No	–	–
Yes	2.24^*^	1.15–4.37

**Notes.**

* *P*-value < 0.05.

Table 3 indicates how the respondents’ trust in medical research affected their willingness to participate in medical research. After demographic factors, including their education level, sex, marital status, and whether they or their household family members held biomedicine-related degrees, were controlled, the following respondents were more likely to participate in medical research: those who expressed trust in doctors that conducted medical research (OR = 1.81, 95% CI [1.28–2.56]), those who agreed that doctors would never ask them to join medical research study that might harm them (OR = 1.45, 95% CI [1.02–2.07]), those who thought that it was safe to be in a medical research study (OR = 2.38, 95% CI [1.68–3.35]), and those who agreed that medical researchers had no selfish reasons for doing research (OR = 1.76, 95% CI [1.22–2.53]).

**Table 3:** The relationship between respondents’ trust in medical research and willingness to participate in medical research.

	Medical research and trust						Willingness to participate in medical research		
	Strongly agree	Agree	Neutral	Disagree	Strongly disagree	Missing	Variables	aOR	95% CI
I completely trust doctors who conduct medical research	67	626	81	560	32	23	Neutral or disagree	–	–
	4.82%	45.07%	5.83%	40.32%	2.30%	1.66%	Agree	1.81^*^	1.28–2.56
Doctors who conduct medical research care only about patients’ interest	24	452	79	761	49	24	Neutral or disagree	–	–
	1.73%	32.54%	5.69%	54.79%	3.53%	1.73%	Agree	1.34	0.95–1.89
A doctor would never ask me to be in a medical research study if the doctor thought it might harm me	60	734	70	456	32	37	Neutral or disagree	–	–
	4.32%	52.84%	5.04%	32.83%	2.30%	2.66%	Agree	1.45^*^	1.02–2.07
It is safe to be in a medical research study	30	432	68	762	61	36	Neutral or disagree	–	–
	2.16%	31.10%	4.90%	54.86%	4.39%	2.59%	Agree	2.38^*^	1.68–3.35
There are some things about medical research that I do not trust at all	17	323	110	846	45	48	Neutral or disagree	–	–
	1.22%	23.25%	7.92%	60.91%	3.24%	3.46%	Agree	0.88	0.60–1.30
Medical researchers have no selfish reasons for doing research studies	56	753	80	438	26	36	Neutral or disagree	–	–
	4.03%	54.21%	5.76%	31.53%	1.87%	2.59%	Agree	1.76^*^	1.22–2.53

**Notes.**

aOR odds ratio adjusted for education level, sex, marital status, and whether the interviewees or their household family members had a biomedicine-related degree CI confidence interval

* *P*-value < 0.05.

## Discussion

In this study, we found that only 12 percent of the respondents would be willing to participate in medical research. In addition, people with a higher education level and those who were married or lived with significant others showed a lower willingness to participate in medical research. In comparison, male respondents, and respondents whose household family members held biomedicine-related degrees or had one themselves, were more likely to participate in medical research. Moreover, after significant demographic variables were controlled, people with a higher level of trust in medical research/researchers demonstrated higher willingness to participate in medical research.

Although our study seems to show low willingness from Taiwanese to participate in medical research, the participation rate was comparable to a household survey in the US (11% among adults) (Davis et al., 2013). In addition, potential financial conflict of interest (FCOI) as described in the research vignette of our study might also influence the participation rate. Previous investigations had shown that people had lower trust in studies with FCOI (Aitken, Cunningham-Burley & Pagliari, 2016; Caulfield et al., 2006; Critchley, Bruce & Farrugia, 2013; Liu & Li, 2017), and low public trust in medical research would in turn reduce the willingness to participate in medical research (Caulfield et al., 2006; DeAngelis, 2000; Gatter, 2003).

Our findings on the importance of trust in medical researchers are consistent with a report from Hall and his colleagues (Hall et al., 2006). They developed two measurement tools to study people’s trust in medical researchers, and they found a positive association between trust in medical researchers and the willingness to participate in hypothetical medical research study. The trust issue must be considered critically. Recently in Taiwan, two prominent biomedical-related researchers were involved in FCOIs, and these incidents aroused significant public concern (Pan, 2017; Chang, 2010). Additional studies are warranted to investigate the impact of these incidents on public trust and participation in medical research.

Notably, the negative effect of education level on participation in medical research is contrary to most of the findings from previous studies conducted in other countries (Bouida et al., 2016; Brewer et al., 2014; Brown & Moyer, 2010; Cobb, Singer & Davis, 2014; Davis et al., 2013; Robinson, Ashley & Haynes, 1996). Our findings are also somewhat different from the results of the three studies conducted in South Korea and Japan, in which the researchers had found no association between education level and participation, and no association between marital status and participation in clinical trials (Chu et al., 2015; Kim et al., 2008; Sugawara et al., 2015). Such discrepancies might be due to the difference between study population of each study. For example, the two studies in Korea and Japan recruited their study participants from hospital patients, and not from general population (Kim et al., 2008; Sugawara et al., 2015). Another explanation might be the trust in doctors. We performed an additional analysis and found that participants with a higher education level expressed lower trust in doctors who conducted medical research (Table S1). This finding was consistent with some previous studies (Freburger et al., 2003; Kayaniyil et al., 2009; O’Malley et al., 2004; Stepanikova et al., 2006). We postulate that our participants with a higher education level had lower trust in doctors; thus, they were less likely to participate in medical research.

It is likewise noteworthy that whether our participants or their close family members held biomedicine-related degrees would positively influence their willingness to join medical studies. This might imply that providing background knowledge would be necessary to increase people’s willingness to participate in medical research. Improving the participants’ awareness regarding why medical research is necessary and how it is conducted might be important (Du et al., 2008). In fact, that is exactly why the National Research Program for Biopharmaceuticals, funded by Taiwanese government, included a clinical trial awareness project (Tsai, 2017).

Our study revealed that women were less likely to participate in medical research than men. The findings of previous studies from other countries were inconsistent on whether sex might influence people’s willingness to participant in medical research. While some studies showed that women expressed lower willingness to participate in medical research because of concerns with potential reproductive adverse effects (Parekh et al., 2011; Wizemann & Pardue, 2001), a review concluded that women were more likely to participate in medical research (Galea & Tracey, 2007). While on the other hand, several nationally representative surveys found no difference on sex for participation rate (Chu et al., 2015; Cobb, Singer & Davis, 2014; Davis et al., 2013; Ewing et al., 2015). Nevertheless, evidence did demonstrate that clinical trials had not always adequately enrolled women (Liu & DiPietro Mager, 2016). For example, according to the US FDA statistics, women made up less than 33% of the participants in phase I trials between 2006 and 2007 (Pinnow et al., 2009). Since there are not enough empirical data on this issue in Taiwan, more studies are required to identify why Taiwanese women may be less willing to participate in medical research.

Our study has the following advantages: (1) limited studies, such as the present study, have examined factors influencing the willingness of Asians to participate in medical research; (2) the present study was conducted as a nationwide survey with a representative sample of adult population in Taiwan; and (3) this study involved face-to-face interviews that were conducted by well-trained interviewers.

Despite the aforementioned merits, the results should be interpreted with caution because of the following limitations: (1) the cross-sectional design of this study created difficulty to distinguish between causes and outcomes, and the reasons behind the factors affecting the willingness to participate in medical research are still undetermined; more follow-up studies, such as examining whether a clinical trial awareness program may lead to better participation rate, are still needed; (2) the clinical trial described in the research vignette was an investigation of a medicinal product, but the respondents might have answered differently if it was another type of medical research; and (3) some medical studies might focus on specific populations, such as children or elders aged above 70 years, which were not included in the survey data we used; the results might be different if the same survey was conducted on the population not included or specified in this study.

## Conclusions

In conclusion, our study identified several demographic factors associated with the willingness to participate in medical research. In addition, trust in medical research seemed to be an independent factor relating to the participation rate. These findings are useful for developing strategies to improve the recruitment of (Asian) participants. Relevant discussions on research ethics and policies, such as the importance of public trust in medical researchers, could also be based on this study.

##  Supplemental Information

10.7717/peerj.4874/supp-1 Table S1 The association between participants’ education level and the trust in doctors who conduct medical research This model was adjusted for age, sex, education level, employment status, personal income, marital status, religion, self-reported health status, place of residence, and whether the interviewees or their household family members had a biomedicine-related degree; *: *P*-vaule < 0.05.

10.7717/peerj.4874/supp-2 Data S1 Genetic research and public opinion: survey interview and database in Taiwan (II)

## Additional Information and Declarations

### Data Availability

The following information was supplied regarding data availability: The raw data is available as a Supplemental File.
