# Supplementary material for: Factors influencing the willingness to participate in medical research: a nationwide survey in Taiwan
Source: PeerJ. 2018 May 31;6:e4874. doi: 10.7717/peerj.4874 (PMC5987748; doi:10.7717/peerj.4874)
Supplement: Table S1 — This model was adjusted for age, sex, education level, employment status, personal income, marital status, religion, self-reported health status, place of residence, and whether the interviewees or their household family members had a biomedicine-related degree; *: P-vaule < 0.05. [file peerj-06-4874-s001.docx]

Table S1. The association between participants’ education level and the trust in doctors who conduct medical research

|  | Odds Ratio | 95%  Confidence Interval |
| --- | --- | --- |
| Education level |  |  |
| Junior high school or below | -- | -- |
| Senior high school or junior college | 0.80 | 0.58-1.10 |
| College or above | **0.65*** | 0.44-0.96 |

This model was adjusted for age, sex, education level, employment status, personal income, marital status, religion, self-reported health status, place of residence, and whether the interviewees or their household family members had a biomedicine-related degree; *: P-vaule<0.05
